# Does Platelet Transcriptome Dysregulation Across the Lewy Body Continuum Mirror Neuronal Dysfunction?

**DOI:** 10.3390/ijms262211169

**Published:** 2025-11-19

**Authors:** Laura Arnaldo, Jorge Mena, David Adamuz, Alex Menéndez, Mònica Serradell, Daniela Samaniego, Carles Gaig, Lourdes Ispierto, Dolores Vilas, Alex Iranzo, Dag Aarsland, Pau Pastor, Katrin Beyer

**Affiliations:** 1 Department of Neuroscience, Research Institute Germans Trias i Pujol, 08916 Badalona, Spain; 2 Departament de Bioquímica i de Biologia Molecular, Facultat de Biociències, Universitat Autònoma de Barcelona, 08193 Barcelona, Spain; 3 Facultat de Medicina, Institut de Neurociències, Universitat Autònoma de Barcelona, 08193 Barcelona, Spain; 4 Unit of Neurodegenerative Diseases, Department of Neurology, University Hospital Germans Trias i Pujol Badalona, 08916 Barcelona, Spain; 5 Sleep Unit, Department of Neurology, Hospital Clínic de Barcelona, Institut d’Investigacions Biomèdiques August Pi i Sunyer (IDIBAPS), Biomedical Research Networking Center on Neurodegenerative Diseases (CIBERNED), 08036 Barcelona, Spain; 6 Centre for Age-Related Medicine (SESAM), Stavanger University Hospital, 4068 Stavanger, Norway; 7 Department of Psychological Medicine, Institute of Psychiatry, Psychology and Neuroscience, King’s College London, London SE5 8AB, UK

**Keywords:** platelet transcriptome, dementia with Lewy bodies, Parkinson’s disease, Alzheimer’s disease, idiopathic REM sleep behavior disorder, RNA sequencing, differential gene expression

## Abstract

Platelets are increasingly recognized as multifunctional cells with roles extending beyond hemostasis to immune regulation, inflammation, and neurodegeneration. Here, we performed RNA-Seq profiling of platelets from patients with idiopathic REM sleep behavior disorder (IRBD), dementia with Lewy bodies (DLB), Parkinson disease (PD), Alzheimer disease (AD), and healthy controls (CTRLs) to explore disease-specific transcriptomic signatures. Across all groups, the RNA class distribution was similar, dominated by mRNAs (78–80%) and long non-coding RNAs (lncRNAs; 15–16%). DLB platelets displayed a reduced proportion of lncRNAs, suggesting an impaired RNA regulation, whereas IRBD concentrated the highest number of disease-specific lncRNAs, half of which were Y-linked, consistent with the male predominance observed in alpha-synucleinopathies. Differential expression analysis (DEA) revealed extensive transcriptomic remodeling in IRBD and DLB, particularly affecting RNA processing, cytoskeletal organization, and platelet activation pathways, while PD and AD showed minimal changes. These findings suggest a progressive impairment of platelet activation and signaling across the DLB continuum, potentially mirroring neuronal dysfunction. The limited transcriptional deregulation in PD may reflect its pronounced biological heterogeneity, consistent with recent multidimensional disease models. Overall, our study highlights platelets as accessible indicators of early and disease-stage-specific molecular alterations in α-synucleinopathies.

## 1. Introduction

Neurodegenerative diseases are a major and growing public health concern, with global cases projected to increase from 54.7 million in 2019 to 152.8 million by 2050, primarily due to aging populations [1]. Alzheimer’s disease (AD) is the most prevalent form, characterized by progressive cognitive decline and neuropathological hallmarks such as β-amyloid (Aβ) plaque accumulation and tau-related neurofibrillary tangles [2]. Dementia with Lewy bodies (DLB), the second-most common degenerative dementia, frequently overlaps with AD both clinically and pathologically, complicating early and accurate diagnosis [3,4]. Misdiagnosis of DLB remains high—up to 80% of cases are initially diagnosed as AD—often leading to suboptimal treatment. While cerebrospinal fluid (CSF) biomarkers, including reduced Aβ42 and elevated tau and neurofilament levels, are well established in AD, their specificity in distinguishing DLB remains controversial [5].

DLB and Parkinson’s disease (PD) are classified as Lewy body disorders (LBD), sharing a common pathophysiology involving the aggregation of α-synuclein [6]. Their underlying mechanisms include mitochondrial and lysosomal dysfunction, iron dysregulation, and neuroinflammation [7]. Importantly, idiopathic REM sleep behavior disorder (IRBD) has emerged as a prodromal phase of LBDs, since up to 91% of patients develop DLB or PD within 14 years [8,9]. Neuropathological and imaging findings in IRBD patients—such as reduced dopamine transporter (DAT) binding and aggregated α-synuclein species in CSF—support the assumption that IRBD is indeed an early synucleinopathy [10,11].

In recent years, blood and its different fractions have been explored as a peripheral biomarker source to support the differential diagnosis of neurodegenerative dementias. Platelets (PLTs), in addition to their crucial function in hemostasis [12], could be bridging blood and brain [13]. PLTs are a primary peripheral source of amyloid precursor protein (APP) in AD, contain α-synuclein, and express various neuronal receptors and enzymes. Additionally, they share biochemical pathways with dopaminergic neurons, including the capacity to store and release neurotransmitters [14]. Despite being anucleate, PLTs also contain diverse classes of RNAs, including mRNAs and miRNAs, along with the corresponding pathways that assure their functionality [15]. Therefore, miRNAs actively regulate mRNA levels [16] and might be responsible for changes in PLT activation capacity. These features highlight their potential role as systemic sensors linking environmental cues to internal physiological states. However, there still exists a significant research gap regarding how platelet transcriptomic alterations reflect central nervous system pathology and whether these molecular changes can reliably distinguish between neurodegenerative disorders. Given recent evidence supporting the role of platelets in neurodegenerative processes, analyzing their transcriptome rather than that of whole blood or PBMCs provides a more targeted and reliable approach, minimizing cellular heterogeneity while capturing biologically meaningful alterations linked to disease mechanisms.

In a recent study we profiled the platelet miRNome in DLB patients compared with controls and identified a DLB-specific biomarker signature [17]. This signature comprises seven miRNAs with decreased expression in DLB compared with controls and AD, discriminating efficiently between the two diseases. In an independent study, we evaluated the same miRNAs in IRBD and found that two of them were already downregulated at this prodromal stage [18].

Taking into account that miRNAs maintain their functionality in PLTs, we hypothesize that the observed reduction of miRNAs in DLB PLTs may lead to the overexpression of several mRNAs in these anucleate cells. Despite evidence supporting the role of platelets as peripheral models of LBD pathophysiology, and our previous findings on the platelet miRNome, the full platelet transcriptome remains unexplored in neurodegenerative diseases. The functional impact of miRNA dysregulation on platelet mRNA expression also remains unknown. Notably, while previous peripheral blood transcriptomic studies have focused on whole blood or PBMCs, this study is the first to apply Next-Generation Sequencing (NGS) to comprehensively profile the platelet transcriptome across these pathologies.

Therefore, the aim of our study was to analyze the PLT transcriptome in patients with DLB, AD, PD, IRBD and cognitively unaffected subjects in order to identify disease-specific expression changes and their associated pathways, providing molecular information required for the discovery of robust diagnostic and prognostic mRNA biomarkers in accessible peripheral blood cells. Additionally, we sought to compare our findings with previous NGS-based transcriptomic studies of PLTs and to explore potential age-related expression patterns.

## 2. Results

### 2.1. Demographic and Clinical Data

Demographic and clinical data of patients are summarized in Table 1. Mean age was similar between DLB patients, IRBD patients and cognitively healthy controls (CTRLs). AD patients were mainly of early-onset, and PD patients were significantly younger. Due to the characteristics of the disease, the male-female ratio was higher in PD and IRBD than in AD and CTRLs. None of the PD patients had developed dementia at the time of sample collection and did not carry any LRRK2 or GBA variant.

### 2.2. Comparison of Gene Expression Profiles Across RNA-Seq Studies

In order to compare our RNA-Seq results with those from previous studies, we searched for RNA-seq studies in PLTs carried out between 2021 and 2025. In their review article, Thibord and Johnson provided a list of 60 RNA-Seq studies conducted between 2011 and 2023 [19]. Over the past two years, some additional PLT RNA-seq studies have been carried out. We retrieved the results from studies involving at least 25 control subjects aged 45 years or older, and three fulfilled the search criteria. Additionally, a study comparing the PLT transcriptome between 20 PD patients and 20 CTRL individuals was also included (Table 2).

#### 2.2.1. CTRLs

**Figure 1:**
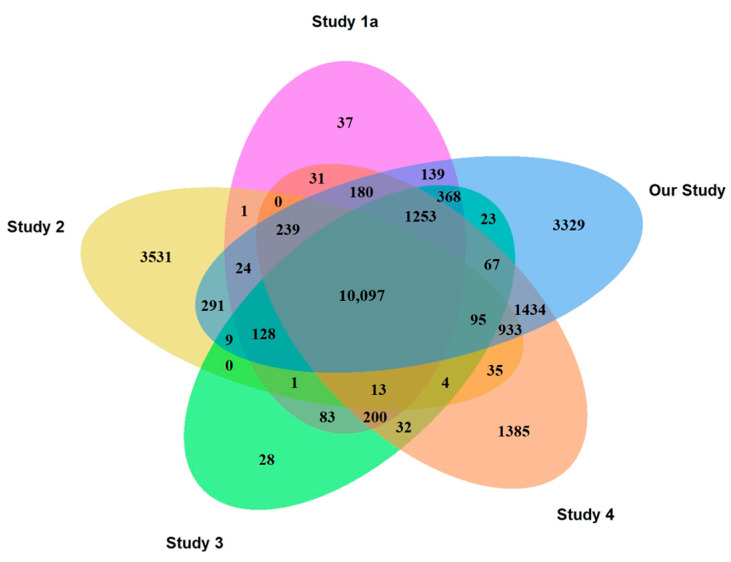
Distribution of the expressed genes in Studies 1a, 2–4 and 5a.

Figure 1 shows the distribution of commonly expressed, overlapping and specific genes identified across the datasets. A total of 10,097 genes were shared among all five studies, corresponding to 78.9% of all genes detected in Study 1a, 65.6% in Study 2, 81.4% in Study 3, 63.1% in Study 4, and 54.3% in CTRLs of our study. In contrast, fewer than 1% of genes were uniquely detected in Studies 1a and 3, whereas 8.7%, 17.9%, and 22.9% of genes were specifically identified in Study 4, our study (Study 5a), and Study 2, respectively. The approximately 3500 genes uniquely expressed in Study 2 were primarily associated with ion channel processes, whereas the 3329 unique genes from our study (Study 5a) were predominantly involved in potassium channel functions.

#### 2.2.2. PD

**Figure 2:**
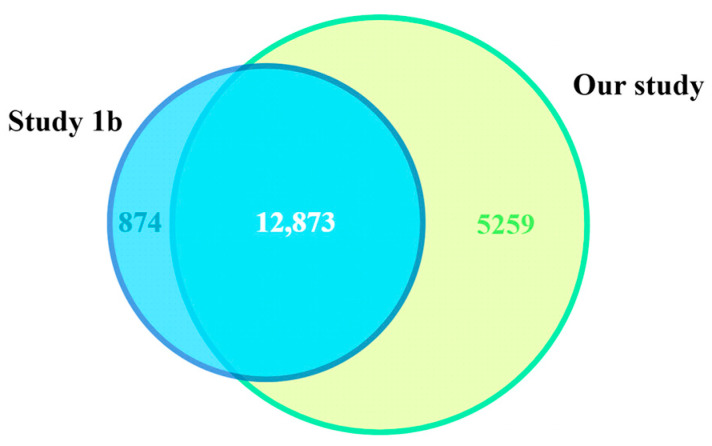
Comparative distribution of expressed genes in Studies 1b and 5b.

The overlap of expressed genes between the two studies that included PD patients (Studies 1b and 5b) is presented in Figure 2. A total of 12,873 genes were commonly expressed across both datasets, corresponding to 93.6% of all genes identified in the PD group of Study 1 and 79% in the PD group of our study (5b). The 5259 genes uniquely expressed in our PD patients were mainly related to ion channel and cytoskeletal motor activity.

### 2.3. Classification of Transcripts Expressed in PLTs

**Figure 3:**
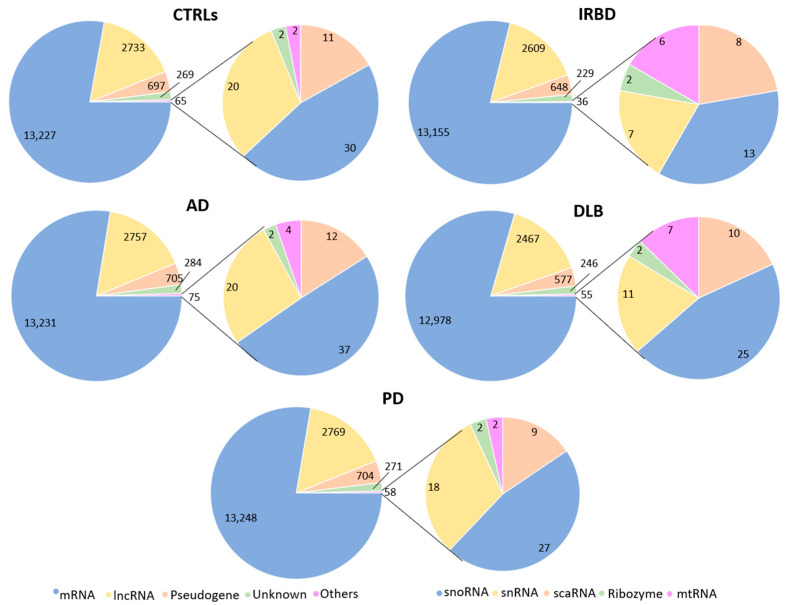
Distribution of mRNAs, lncRNAs, pseudogene RNAs, scaRNAs, snoRNAs, snRNAs, ribozymes and mtRNAs in the 5 groups included in this study.

The distribution of five major RNA classes identified for each group is shown in Figure 3. Whereas 78–80% were protein-expressing genes, 15–16% were long non-coding RNAs (lncRNAs), 4% were pseudogenes and 1–2% were unknown transcripts. The group of minor RNAs, composed of small Cajal body-specific RNAs (scaRNAs), small nucleolar RNAs (snoRNAs), small nuclear RNAs (snRNAs), ribozymes and mitochondrial RNAs (mtRNAs), represented less than 1% of all RNAs.

#### 2.3.1. Long-Non-Coding RNA (lncRNA)

**Figure 4:**
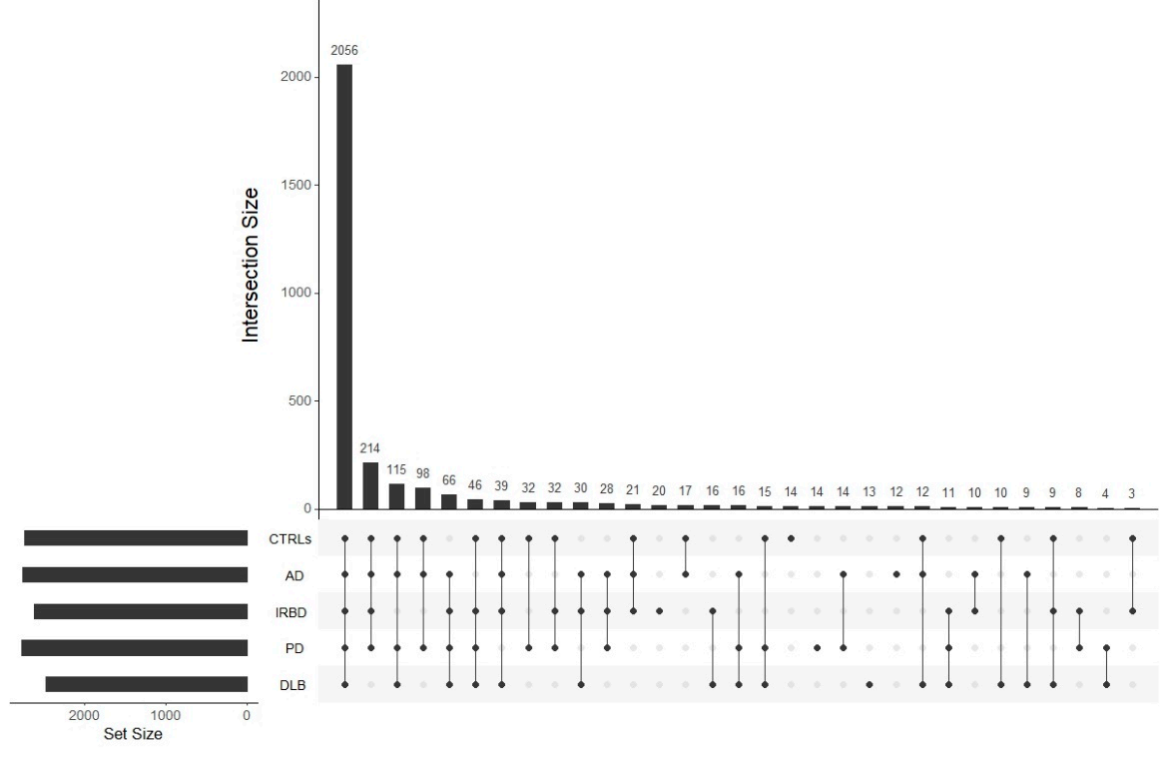
Distribution of lncRNA genes in the different groups.

The distribution of lncRNA differed among the five groups (*p* = 0.0064), and the relative amount of lncRNAs was smaller in DLB compared with CTRLs, AD and PD (*p* = 0.0063, 0.0023 and 0.0001, respectively). The relative amount of lncRNA was slightly, but not significantly lower in IRBD compared with CTRLs, AD and PD. However, the number of group-specific lncRNAs was similar in all groups, 13 in DLB, 20 in IRBD, 14 in PD, 12 in AD and 14 in CTRLs (Figure 4). Among those, whereas in DLB the majority (92.3%) were uncharacterized, only 50% were uncharacterized in IRBD. Group-specific lncRNA genes were distributed across all chromosomes, except for IRBD, where 50% of lncRNA genes were located on chromosome Y (Appendix A.1).

#### 2.3.2. Minor RNAs

**Figure 5:**
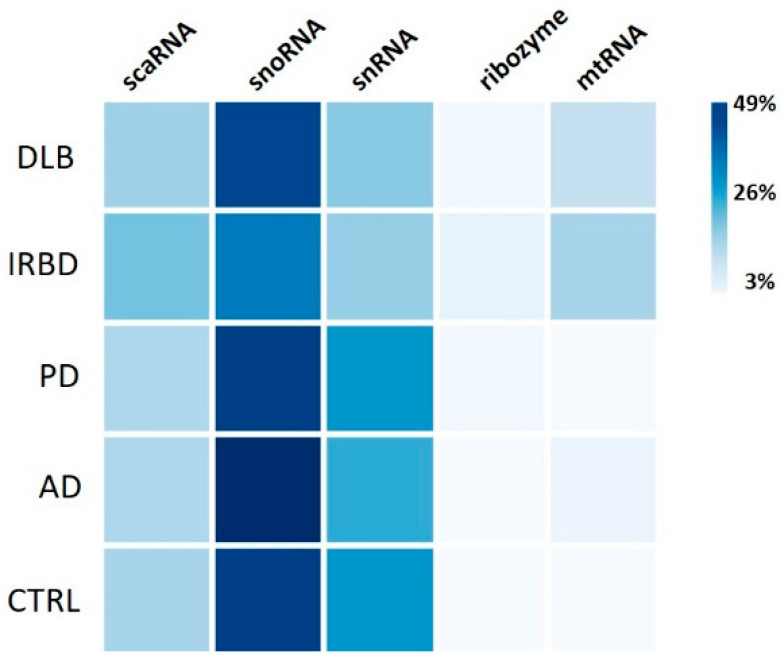
Percentage distribution of the five minor RNA groups in the diseases and controls.

The analysis of minor RNAs revealed that most of them were snoRNAs (36–49%), followed by snRNA (19–31%) and scaRNAs (16–22%; Figure 1). Two ribozymes, RMRP and RPPH1, were present in all groups, representing between 3% and 5% of minor RNAs, and mtRNAs represented between 13% and 17% in DLB and IRBD, respectively, but only between 3% and 5% in PD, AD and CTRLs (Figure 5, Appendix A.2).

**Figure 6:**
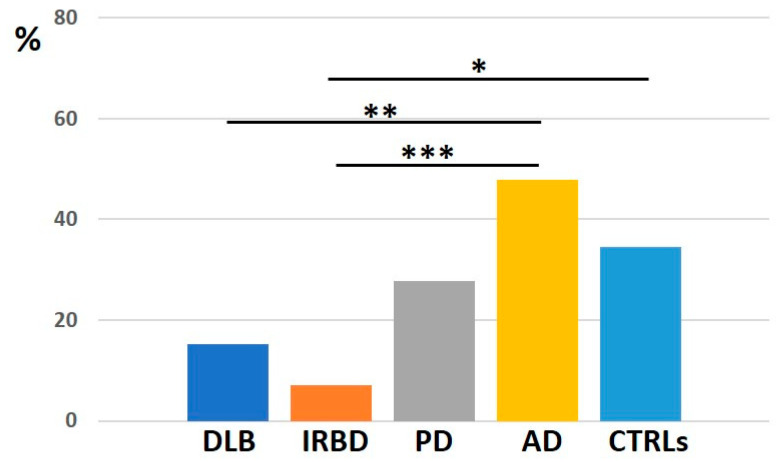
Distribution of nuclear RNAs (scaRNAs, snoRNAs and snRNAs) across the five datasets, illustrating group-specific transcripts. Group-specific RNAs are defined as those detected in one or more, but not all, of the analyzed groups. *, *p* < 0.05, **, *p* < 0.01, ***p < 0.001.

Whereas 61.5% of scaRNAs were found in all groups, only 24.5% of snoRNAs, 20% of snRNAs and 28.6% of mtRNAs were commonly expressed (Appendix A.3). When dividing the minor RNA groups into nuclear and mtRNAs, we found that IRBD expressed less specific nuclear RNAs compared with CTRLs and AD (*p* = 0.042 and *p* = 0.0038, respectively), and DLB compared with AD (*p* = 0.0071; Figure 6). On the contrary, both IRBD and DLB contained more specific mtRNA (66.8 and 71.4%, respectively) compared especially to PD and CTRLs, which expressed only common mtRNAs (Appendix A.3).

### 2.4. Differential Gene Expression, Gene Ontology (GO) Enrichment and KEGG Pathway Analysis

First, a post hoc power sensitivity analysis was conducted to determine the power of the study to detect a biologically relevant effect. The calculated statistical power was 96.8% and 97.8%, respectively.

**Figure 7:**
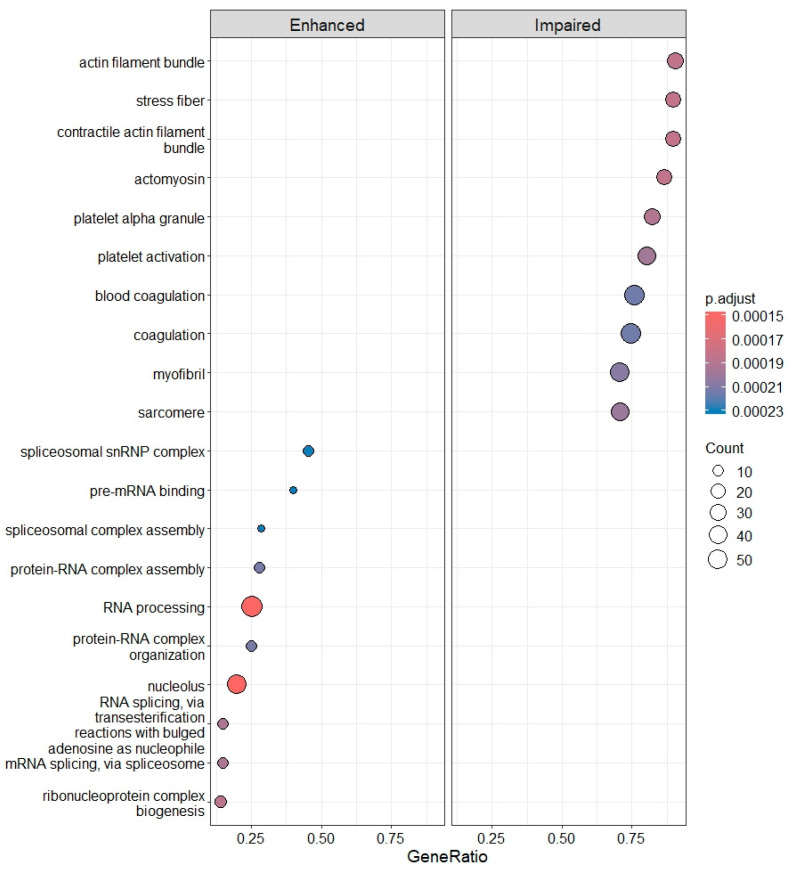
Enhanced and impaired biological processes identified by gene ontology (GO) enrichment in IRBD compared with CTRLs.

In IRBD, 4690 differentially expressed genes (DEGs) were identified compared with CTRLs. Of these, 2568 (54.7%) showed increased and 1493 (45.3%) decreased expression. To determine which biological processes were positively or negatively affected, DEGs were subjected to Gene Ontology (GO) enrichment analysis. As a result, we observed an increase in the spliceosome and several mechanisms involved in RNA processing, and a decrease in processes related to PLT activation function. These specifically included the impairment of the actin cytoskeleton and PLT alpha-granules (Figure 7).

**Figure 8:**
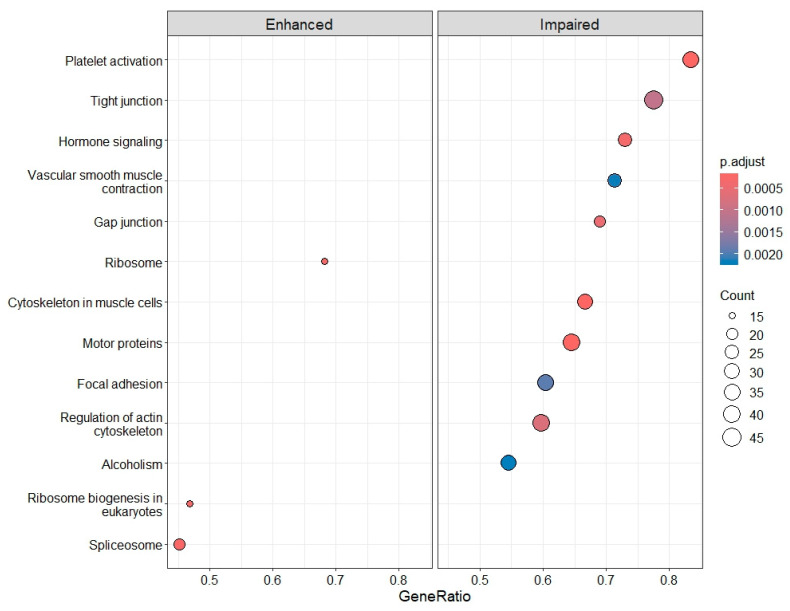
Enhanced and impaired KEGG pathways in IRBD compared with CTRLs.

To further understand the functional consequences of gene overexpression or diminution, both gene lists were studied by Kyoto Encyclopedia of Genes and Genomes (KEGG) pathway analysis. Whereas the spliceosome was upregulated (Appendix B.1), an overall moderate downregulation of PLT activation-related processes was observed again (Appendix B.2). The latter included the diminution of junction proteins and impaired actin cytoskeleton regulation (Figure 8 and Appendix B.3).

**Figure 9:**
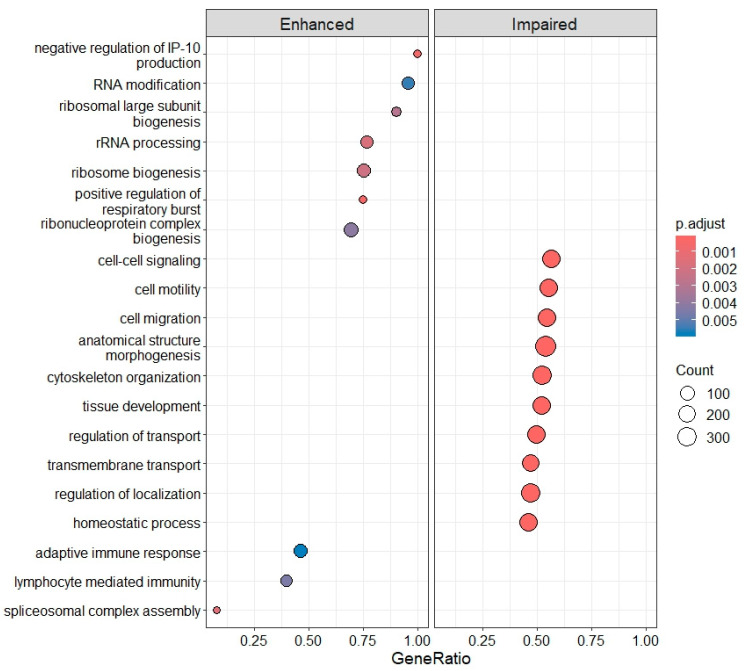
Enhanced and impaired biological processes identified by GO enrichment in DLB compared with CTRLs.

In DLB, 6475 DEGs were found, of which 4036 were increased (73.5%) and 1778 decreased (26.5%). These DEGs were involved in 114 different biological processes identified by GO enrichment, with the most significantly enhanced processes related to ribosomal biogenesis, RNA modification, and the immune response, specifically involving adaptive immune response and lymphocyte-mediated immunity. In contrast, PLT activation (cytoskeleton organization, cell-to-cell signaling), adhesion (cell motility, regulation of localization) and aggregation (anatomical structure morphogenesis, tissue development) were markedly impaired, involving between 200 and 300 genes each (Figure 9).

**Figure 10:**
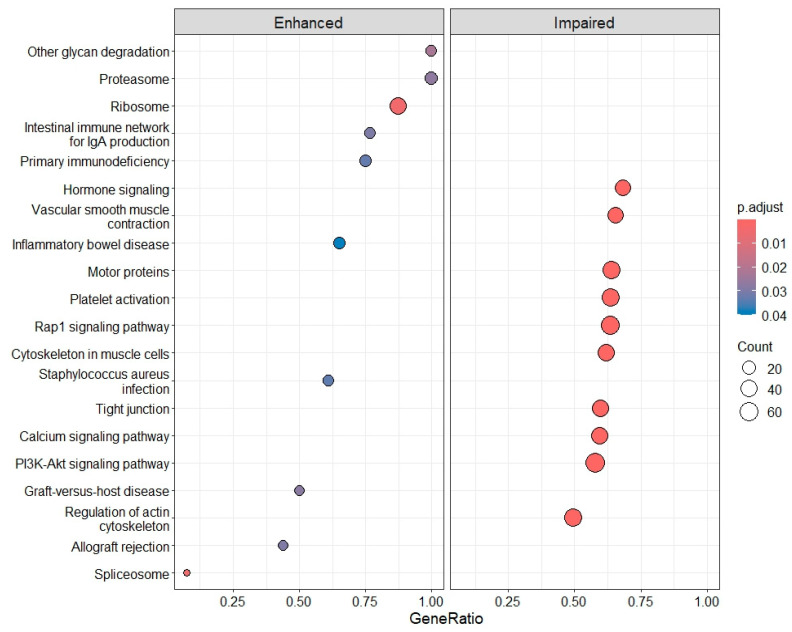
Enhanced and impaired KEGG pathways in DLB compared with CTRLs.

KEGG pathway analysis revealed an enhancement of pathways related to the ribosome, and the impairment of pathways related to PLT activation, including Ca^2+^- and Rap-signaling pathways and the regulation of the actin cytoskeleton, involving approximately 40 to 50 genes per process (Figure 10, and Appendix B.2, Appendix B.3 and Appendix B.4, respectively).

In PD patients, only 23 genes were deregulated (1 gene (GPC6) increased and 22 decreased) and enriched GOs were related to peroxidase and oxygen activity, and enhanced pathways related to ribosomes. Finally, in AD patients, only 12 genes were deregulated (7 increased and 5 decreased). Most of these genes were enriched for GO terms related to microtubule minus-end binding and enoyl-CoA hydratase activity.

## 3. Discussion

In this study, we analyzed the PLT transcriptome in synucleinopathies including DLB, PD and one of their prodromal forms, IRBD, as well as in AD, and compared these groups with CTRLs. An increasing number of studies have begun to address PLT physiology, including their transcriptome, as PLTs are growingly recognized as multifunctional cells that extend far beyond their traditional role in hemostasis. They actively participate in inflammation, immune regulation, angiogenesis, and vascular integrity [24,25]. In recent years, this multifunctionality has linked PLTs to a wide spectrum of systemic and neurological diseases, since they contain abundant α-synuclein, APP and β-amyloid, and share key molecular and signaling machinery with neurons [26]. These shared features suggest that PLT function may mirror or even influence neuronal processes underlying neurodegeneration [27]. Thus, we sought to characterize the composition of the PLT transcriptome comprising five major RNA classes. Additionally, we wanted to identify altered molecular pathways in PLTs that could reflect disease-specific mechanisms of α-synucleinopathies and potentially serve as peripheral indicators of early or ongoing neurodegenerative changes.

### 3.1. Comparison of PLT RNA-Seq Studies

When comparing our RNA-Seq data with the other four studies, our dataset showed the highest number of expressed genes, particularly relative to Studies 1 and 3. This difference likely reflects greater sequencing depth in our study, as more reads per sample increase the detection of low-abundance transcripts. Our libraries were sequenced to ~50 million reads per sample, whereas this information was not reported for Studies 1–4. Correspondingly, genes uniquely detected in our PD samples but absent in Study 1b were predominantly low-expression genes.

Read length, sequencing strategy, and library preparation may also contribute to the detection of more genes. We used 150 bp paired-end reads, which improve alignment accuracy in repetitive regions such as pseudogenes and gene families. In contrast, three of the other studies used 100 bp single-end reads (except Study 4, which used paired-end reads), likely leading to more ambiguous alignments and fewer detected genes.

Additionally, we used the Illumina Stranded Total RNA Prep with Ribo-Zero Plus kit, which includes rRNA depletion and enhanced detection of low-abundance transcripts. The SMARTer Ultra Low RNA Kit used in Studies 2–4 lacks this depletion step, thus rRNA dominates the library, reducing sensitivity for other RNA species. Although the kit for library preparation was not reported for Study 1, the use of fragmented mRNA during library preparation could also explain the lower number of expressed genes compared to the other studies.

### 3.2. The Composition of the PLT Transcriptome

Across all five groups, IRBD, DLB, PD, AD and CTRLs the overall distribution of RNA classes was comparable, with mRNAs accounting for 78–80% of transcripts, lncRNAs for 15–16%, pseudogenes for approximately 4%, and 1–2% classified as unknown transcripts. This composition indicates that, in PLTs, protein-coding transcripts dominate the RNA landscape, while lncRNAs constitute a smaller but potentially functionally relevant subset. Additionally, it reflects the limited transcriptional activity of these anucleate cells and their reliance on mRNAs and regulatory RNAs inherited from megakaryocytes.

Interestingly, we observed a lower proportion of lncRNAs in DLB compared with the other groups. This reduction may indicate disease-specific alterations in the regulatory RNA repertoire of PLTs, potentially reflecting broader dysregulation in RNA processing, stability, or megakaryocyte-derived transcript packaging in DLB. Although lncRNAs are less abundant in PLTs than in nucleated cells, previous studies have shown that they exert key regulatory roles in formation, activation, and intercellular communication [28,29]. Large-scale transcriptomic analyses across human tissues have reported that lncRNAs can outnumber protein-coding genes and display high tissue specificity, whereas in PLTs an inverse trend is observed, with approximately fivefold more protein-coding genes than lncRNAs [30,31]. The reduced lncRNA fraction in DLB, therefore, may represent a loss of specific regulatory RNAs or an altered balance between coding and non-coding components of the PLT transcriptome, potentially mirroring disease-related changes in cellular homeostasis and RNA metabolism. Further studies integrating lncRNA expression with functional PLT phenotypes and disease severity could help to clarify whether these transcriptomic shifts have diagnostic or mechanistic significance in DLB and related synucleinopathies.

Among all groups, IRBD exhibited the highest number of disease-specific lncRNAs, with half (10 of 20) located on the Y chromosome. This striking enrichment may reflect sex-linked transcriptional regulation, consistent with the strong male predominance of IRBD and related synucleinopathies [32]. The presence of Y-linked lncRNAs in PLTs could therefore indicate early, sex-specific molecular alterations associated with prodromal stages of α-synuclein-related neurodegeneration.

### 3.3. Deregulation of Gene Expression in PLTs

When analyzing DEGs across the four disease groups compared with CTRLs, DLB exhibited the highest number of DEGs (>6400), followed by IRBD (4690). In contrast, both PD and AD showed fewer than 25 DEGs each. This distribution reveals two distinct transcriptomic response patterns: (1) a high-extent deregulation in IRBD and DLB, and (2) a low-extent response in PD and AD. These findings suggest two underlying biological behaviors—first, a progressive molecular impairment and shared pathway dysregulation within the LBD spectrum (IRBD–DLB continuum); and second, greater group heterogeneity in PD and AD, making it difficult to define disease-specific molecular signatures.

#### 3.3.1. IRBD and DLB: Progressive Molecular Impairment and Shared Pathway Dysregulation

The extensive transcriptomic remodeling observed in IRBD, which is even more pronounced in DLB, suggests that IRBD may capture a prodromal phase marked by active molecular adaptation (4690 DEGs), whereas DLB reflects progression toward sustained inflammatory and translational activation (>6400 DEGs), consistent with advanced synucleinopathy. In IRBD, DEGs were strongly enriched in upregulated RNA processing pathways, particularly spliceosome-mediated pre-mRNA splicing, suggesting increased post-transcriptional activity in PLTs. This may reflect an adaptive response or early dysregulation of RNA maturation processes. Notably, specific nuclear RNAs were reduced compared with CTRLs and AD, indicating a possible imbalance between spliceosome assembly and RNA substrate availability. Such alterations could mirror compensatory or stress-induced changes in RNA metabolism, consistent with early molecular disturbances preceding over neurodegeneration.

Aberrant RNA processing has been increasingly linked to synucleinopathies, where α-synuclein interacts with RNA-binding proteins such as TDP-43, FUS, and hnRNPs, disrupting splicing, RNA stability, and translation [33,34]. The activation of spliceosomal pathways and concurrent nuclear RNA reduction in IRBD PLTs may therefore represent systemic manifestations of these neuronal processes, highlighting altered RNA metabolism as an early hallmark of disease [35].

Additionally, pathway analysis in IRBD revealed the impairment of PLT activation affecting the actin cytoskeleton, junction proteins and alpha-granule release, indicating early functional alterations that may compromise PLT responsiveness. Strikingly, in DLB, PLT activation mechanisms showed an overall impairment extending from the first activation steps to mechanisms related to adhesion and aggregation. At the same time, key intracellular signaling routes, including Ca2+- and Rap-dependent pathways, were also downregulated, indicating impaired signal transduction essential for PLT activation and integrin-mediated responses [36,37]. Taken together, these data point to a progressive loss of PLT activation capacity from IRBD to DLB, suggesting that cytoskeletal and signaling dysfunction in PLTs may mirror comparable alterations in neuronal and synaptic physiology characteristic of advancing synucleinopathy. However, whether these changes mirror impaired synaptic function needs to be further studied.

This progressive impairment of PLT activation and cytoskeletal regulation observed from IRBD to DLB could be linked to the physiological and pathological roles of α-synuclein. Under normal conditions, α-synuclein participates in vesicle trafficking, membrane curvature sensing, and actin cytoskeleton dynamics—processes essential for both neurotransmitter release in neurons and granule secretion in PLTs [38,39,40]. But, additional research should be carried out to determine whether the reduced activation, adhesion, and aggregation capacity, as found in our transcriptomic study, are secondary to α-synuclein dysregulation or aggregation.

Additionally, in our study, platelets from DLB patients showed a marked upregulation of immune-related pathways, particularly those linked to adaptive and lymphocyte-mediated responses. Similarly to findings in inflammatory disorders such as COVID-19, sepsis, and systemic lupus erythematosus, this suggests that platelets actively modulate immune processes through cytokine release and interactions with lymphocytes and endothelial cells [41]. The presence of comparable immune activation signatures in DLB indicates that platelet–immune crosstalk may contribute to neurodegenerative mechanisms, potentially mediated by platelet-derived α-synuclein or inflammatory signaling. Overall, these results indicate that platelets could represent dynamic immunomodulatory cells connecting peripheral immune alterations with central pathology in LBD, a hypothesis that needs further corroboration.

#### 3.3.2. PD and AD: Disease Heterogeneity

In our PLT RNA-Seq data, the striking contrast between a lack of transcriptional signal in PD and AD versus the large signal in IRBD and DLB highlights the heterogeneity within neurodegenerative diseases and underscores the potential for masked subtype-specific signatures. Particularly, this limited transcriptional response in PD may reflect the substantial biological heterogeneity of the disease. As shown by the recently proposed SynNeurGe framework, PD encompasses multiple interacting dimensions—α-synuclein pathology (S), neurodegeneration (N), and genetic predisposition (G)—which combine to produce diverse clinical phenotypes (C) [42]. This multidimensional model underscores that individuals clinically diagnosed with PD may represent distinct molecular subtypes with variable synuclein burden, neurodegenerative progression, and genetic background. Consequently, pooled transcriptomic analyses may obscure subtype-specific signatures, masking expression changes that are more evident in biologically homogeneous groups such as IRBD or DLB.

Comparable heterogeneity has been demonstrated in AD. In a recent large-scale study, five molecular AD subtypes based on CSF proteomic profiles have been identified. These were defined, respectively, by hyperplasticity, innate immune activation, RNA dysregulation, choroid plexus dysfunction, and blood–brain barrier impairment [43]. These subtypes differ in genetic risk factors, cortical atrophy patterns, and clinical trajectories. Similarly, a meta-analysis integrating neuropathological and neuroimaging data delineated four biological subtypes—typical, limbic-predominant, hippocampal-sparing, and minimal atrophy AD—each with distinct regional tau distribution, demographic associations, and disease progression patterns [44]. All together, these findings reinforce that both PD and AD encompass multiple mechanistic trajectories rather than a single linear disease continuum.

Within this framework, the absence of major transcriptomic deregulation in AD PLTs may indicate that PLTs do not capture all biological pathways involved across AD subtypes. Given our limited sample size, subtype-specific alterations may have been masked in the current analysis. Correspondingly, it could be expected that PLTs of patients with the AD subtype characterized by blood–brain barrier impairment would indeed show important transcriptomic changes [45,46].

### 3.4. Future Biomarker Development

Our findings—demonstrating altered gene expression profiles in platelets, an easily accessible biofluid—in DLB and IRBD provide strong evidence for their potential translation into clinically useful biomarkers. To advance this translational pathway, we are currently finalizing differential expression analyses among disease groups, including both synucleinopathies (DLB and PD), IRBD, and DLB versus AD, to delineate pathology-specific transcriptional signatures. Building on the RNA-seq data, which revealed extensive dysregulation in pathways such as the spliceosome, ribosome, platelet activation, and immune response, we are now focusing on defining a core, clinically actionable gene signature. This will involve prioritizing top-ranking DEGs with the highest effect sizes and lowest variability, particularly those belonging to the most discriminative pathways, followed by orthogonal validation using qPCR to ensure robustness and technical reproducibility.

To ensure clinical reliability and generalizability, we plan to validate this core signature in an independent, large, and clinically well-characterized cohort. The validated qPCR data will then be used to develop predictive models—such as logistic regression or machine learning approaches—optimized for diagnostic and prognostic applications. Specifically, these models aim to distinguish DLB from AD and to predict phenoconversion in IRBD patients who progress to DLB or PD. Translating the platelet RNA signature into a routine clinical assay will also require addressing key optimization procedures and establishment of clinically relevant cut-off values based on model sensitivity and specificity. Together, these efforts are expected to facilitate the development of a robust and clinically implementable diagnostic and prognostic assay for synucleinopathies.

## 4. Materials and Methods

### 4.1. Source of PLT Samples

In total, 64 individuals were prospectively recruited at the Neurodegenerative Disease Unit of the Neurology Department from the Hospital Universitari Germans Trias i Pujol (HUGTP; Badalona, Barcelona, Spain) and Sleep Unit of the Neurology department from the Hospital Clinic de Barcelona (Barcelona, Spain). The cohort included individuals divided into five groups. The four patient groups were: 12 DLB patients who fulfilled criteria for probable DLB [3], 12 PD patients diagnosed according to the UK PD Society Brain Bank criteria [47], 12 IRBD patients who fulfilled diagnostic criteria in subjects reporting nightmares and dream-enacting behaviors in whom nocturnal video-polysomnography showed increased electromyographic activity in REM sleep [48], and 14 AD patients fulfilling criteria for probable AD (National Institute on Aging–Alzheimer’s Association criteria) [49]. Group five was composed of 14 cognitively unaffected controls (CTRLs), mainly non-blood relatives of the patients without a family history of neurological disorders, suggestive symptoms of IRBD or any symptoms or signs indicating parkinsonism or cognitive impairment.

The study was approved by the HUGTP Ethical Committee for Clinical Investigation (PI-22-024). All participants or their legal guardians signed written informed consent according to the Declaration of Helsinki [50].

### 4.2. PLT Obtaining and RNA Purification

Na-citrate Vacutainer tubes (BD, Plymouth, UK) were used to collect blood samples. They were processed by two consecutive centrifugation steps within the first four hours after blood extraction to avoid expression changes due to PLT activation. First centrifugation was performed at 200× *g* for 15 min to obtain PLT-rich plasma, and the following centrifugation was carried out at 2500× *g* for 15 min to obtain PLT-rich pellets (PRP). PRP was stored at −80 °C until RNA purification and thawed on ice before processing. Total RNA isolation was performed using the mirVana^TM^ miRNA Isolation Kit (ThermoFisher, Waltham, MA, USA), and isolated RNA samples were stored at −80 °C until expression analysis.

### 4.3. Total RNA Discovery by Next-Generation Sequencing (NGS)

The concentration of total RNA samples was measured by Qubit and 15 ng from each sample were adjusted to a final volume of 6.5 µL. Samples were pooled in pairs. Pooling was done for samples with similar concentrations, to assure similar RNA proportions and avoid confounding enrichment. Quality control and size distribution of the pools was assessed with the 4200 TapeStation System (Agilent Technologies, Santa Clara, CA, USA) using High Sensitivity RNA ScreenTape (Agilent, Santa Clara, CA, USA). Of the pooled sample pairs, only 5 had RIN values between 5.5 and 6.5; the rest of them presented RIN values higher than 6.5.

From each pool, 11 µL was used for library preparation by Illumina Stranded Total RNA Prep, Ligation with Ribo-Zero Plus for Illumina (Illumina, San Diego, CA, USA) following the manufacturer’s instructions. Amplification cycles were adjusted according to the estimated RNA concentration. Individual libraries were subjected to quality analysis and quantification using a D1000 ScreenTape (Agilent Technologies, Santa Clara, CA, USA). If the library profile was not acceptable, a repurification step was performed. All libraries were adjusted to 8 nM in a final volume of 5 µL and were all pooled together. The library pool was finally assessed by High Sensitivity D1000 ScreenTape (Agilent Technologies, Santa Clara, CA, USA).

Clustering and sequencing were performed in an Illumina Sequencer (NovaSeq6000, Illumina, San Diego, CA, USA). Samples were sequenced to ~50 million (M) 2 × 150 bp paired-end. Sample quality was checked using FastQC and MultiQC tools (version 0.12.0 and 1.14, respectively) [51,52]. The obtained FastQ raw data was analyzed as follows: (1) the TrimGalore tool (version 0.6.10) was used to remove the adapter sequences from the reads and the base pairs with a Phred score under 20 [53]; (2) paired RNAseq reads were aligned to the Gencode GRCh38.p109 human reference genome using STAR (version 2.7.10b) [54]; (3) the Salmon tool (version 1.4.0) was used for quantifying the expression of each transcript [55]; (4) the matrix count was generated with tximport (version 1.30.0) [56]; (5) the total count of reads was normalized using the median of ratios method from DESeq2 package (version 1.45.1) [57]. Steps 1 to 3 were performed from the Ubuntu terminal (Linux), while steps 4, 5, and the following analysis were conducted using R software (version 4.3.2).

### 4.4. Sequencing Data Analysis

For NGS expression analysis, transcripts with a minimum of 10 reads per sample were considered expressed. Gene biotype classification (mRNA, pseudogene, lncRNA, scaRNA, snRNA, snoRNA, rRNA, and mitochondrial RNA) of the genes that were expressed in the different groups (AD, IRBD, DLB, PD, and CTRLs) was obtained using the getBM function from the biomaRt package (version 2.58.2).

Differential expression analysis (DEA) was performed using the Wald test, and *p*-values were corrected by the Benjamini–Hochberg method with DESeq2, establishing significance as an adjusted *p*-value lower than 0.05. A post hoc power sensitivity analysis was performed assuming a Fold-Change of 2 and using the RNASeqPower package (version 1.42.0).

### 4.5. Comparison of RNA Expression Between RNA-Seq Studies

RNA-seq data from four different studies analyzing the whole PLT transcriptome was retrieved from the NCBI Gene Expression Omnibus (GEO) and NCBI Sequence Read Archive (SRA) databases: PRJNA732990, PRJNA732803, GSE183635, PRJNA668820 and PRJNA737596, (defined in results as studies 1a, 1b, 2, 3 and 4, respectively). For the GSE183635 study, raw data counts were obtained directly from its repository. For PRJNA732990, PRJNA732803, PRJNA668820 and PRJNA737596 studies, FastQ files were acquired. As these studies used paired-end sequencing, FastQ files of all samples were processed similarly to those in our study, using the Salmon tool for quantifying the expression of each transcript and the tximport function for generating the matrix count. Only the expression of control individuals from each study was considered in the comparative analysis, except for Study 1 where PD samples were also obtained. In all samples, only transcripts with at least 10 reads were kept. A Venn diagram (VennDiagram package, version 1.7.3) was obtained to visualize the expression of overlapping genes among studies.

### 4.6. LncRNA and Minor RNA Distribution Analysis

The distribution of RNA biotypes and four groups of minor RNAs (scaRNAs, snoRNAs, snRNAs and mtRNAs) was analyzed by comparing all five groups. Pairwise comparison between groups was carried out using chi-square and Fisher’s exact test. The results were corrected for multiple testing by the Bonferroni method. Significance was set at 0.05.

### 4.7. Gene Ontology (GO) Enrichment and KEGG Pathway Analysis

Gene Set Enrichment Analysis (GSEA) of transcripts differentially expressed between the four diseases (IRBD, AD, DLB and PD) and CTRLs was performed using the enrichGO and gseDO function from clusterProfiler (verison 4.10.1) and DOSE packages (version 3.28.2), respectively; and KEGG pathway analysis using gseKEGG function from DOSE package. Dotplot function from DOSE package was used to generate dotplots. A *p*-value below 0.05 was considered a significant enrichment.

GO enrichment was also analyzed for the genes specifically expressed in CTRL individuals from each of the five different RNAseq studies and in PD patients from Study 1b and our study.

## 5. Conclusions

In summary, our study provides the first comprehensive comparison of PLT transcriptomes across major neurodegenerative disorders, revealing disease- and stage-specific molecular alterations. PLTs from IRBD and DLB patients displayed extensive transcriptomic remodeling, encompassing dysregulation of RNA processing, cytoskeletal organization, and activation pathways. These findings suggest that systemic RNA metabolism and PLT signaling mechanisms may mirror early and progressive stages of α-synucleinopathy. In contrast, the limited number of DEGs in PD supports the growing view that PD represents a highly heterogeneous disorder, in which diverse molecular subtypes may obscure shared transcriptomic signatures at the group level.

The absence of significant deregulation in AD PLTs further underscores that not all central pathological processes are reflected peripherally, particularly given the molecular heterogeneity of AD subtypes. Nonetheless, our results highlight the potential of PLT transcriptomics to reveal peripheral molecular correlates of neurodegeneration and identify early systemic changes in prodromal synucleinopathies such as IRBD. Future studies integrating multi-omic PLT profiling with clinical, imaging, and genetic data will be essential to validate these findings and to determine whether specific PLT transcriptomic signatures could serve as accessible biomarkers of disease onset, subtype, or progression across neurodegenerative disorders.

## Figures and Tables

**Table 1:** Demographic and clinical data of the participants of the study.

	DLB (*n* = 12)	PD (*n* = 12)	IRBD (*n* = 12)	AD (*n* = 14)	CTRL (*n* = 14)	*p* ^1^
Mean age, y ^2^ (age range, y)	74.1 (64–85)	66.9 (44–87)	74.5 (65–89)	68.8 (60–80)	71.3 (61–86)	0.017
Gender (male/female ratio)	7/5	8/4	9/3	7/7	7/7	0.069
Duration ^3^, years (range)	5.7 (2.1–10.6)	15.2 (4.9–23.7)	8.9 (2.5–18.2)	5.2 (0.8–8.0)		
MMSE ^4^, mean (range)	15.3 (3–27)	n.a. ^5^		19.9 (5–28)	-	0.189
UPDRS-III ^6^, mean (range)	-	20.9 (5–39)		-	-	-
GDS fast ^7^, mean (range)	-	-		4.1 (3–6)	-	-
Parkinsonism, n (%)	10 (83.3%)	-	-	-		-
Positive DAT imaging, n (%)	11 (91.6%)	-	-	-		-

^1^ *p*, *p*-value obtained by the Kruskal–Wallis test; ^2^ y, years old; ^3^ duration, disease duration from disease onset to sample obtaining; ^4^ MMSE, Mini-Mental State Examination; ^5^ n.a., not applicable, since patients had no signs of cognitive impairment, thus MMSE was not carried out; ^6^ UPDRS-III, Unified Parkinson’s Disease Rating Scale Part III; ^7^ GDS, Global Deterioration Scale.

**Table 2:** Platelet RNA-Seq studies between 2021 and 2025, with more than 25 control individuals.

Study	Year	Accession Number	Samples (n)	Age ^1^	Expressed Genes ^2^
1a	2021 [20]	PRJNA732990	20 CTRLs	49.2 (21–75)	12,794
1b	2021 [20]	PRJNA732803	20 PD	67.1 (50–86)	13,747
2	2022 [21]	GSE183635	316 CTRLs	55.4 (18–86)	15,402
3	2022 [22]	PRJNA668820	56 CTRLs	47.8 (n/a)	12,401
4	2022 [23]	PRJNA737596	190 CTRLs	54.6 (31–72)	15,998
5a	2025	Our study	14 CTRLs	71.3 (61–86)	18,609
5b	2025	Our study	12 PD	66.9 (44–87)	18,132

^1^ Mean age (age range). n/a—not available. ^2^ Number of genes with more than 10 reads in all samples of each study.

**Figure A1:**
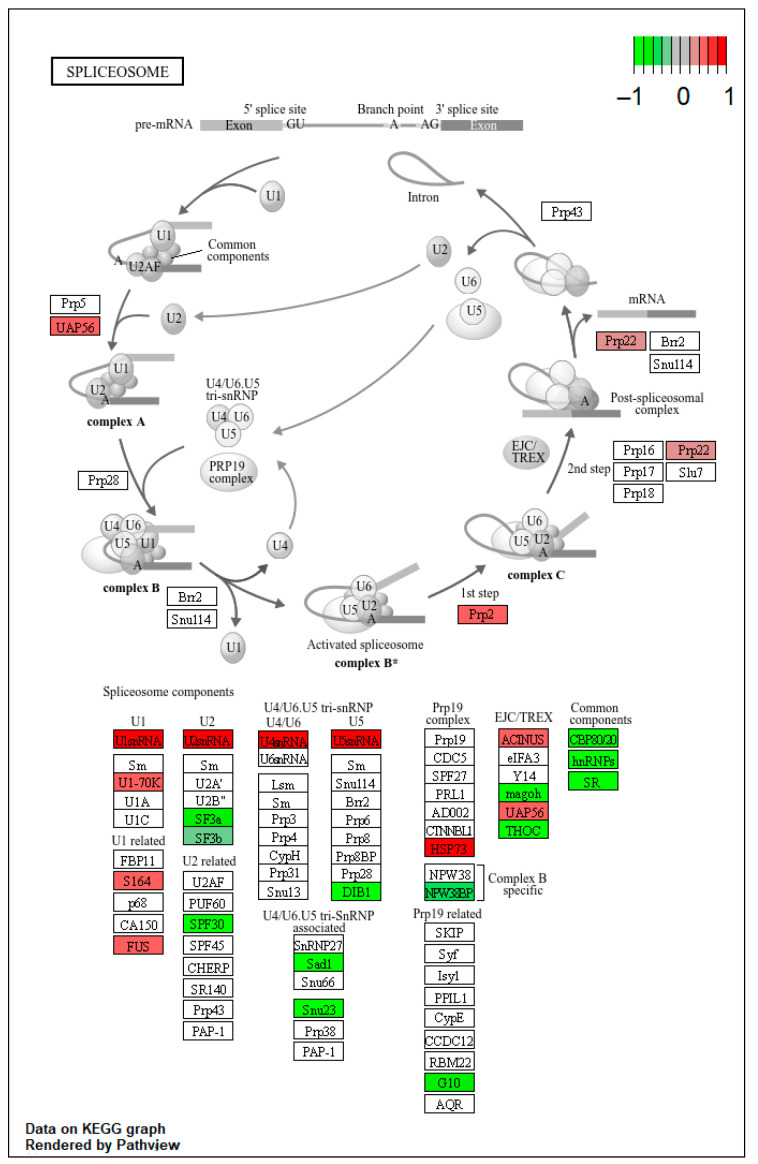
Spliceosome components with highlighted DEGs comparing IRBD vs. CTRLs, where all snRNAs (except U6) involved in the spliceosome pathway showed an increased expression in IRBD and thus, an enhancement of the pathway is expected. Decreased genes are marked in green and increased genes in red.

**Figure A2:**
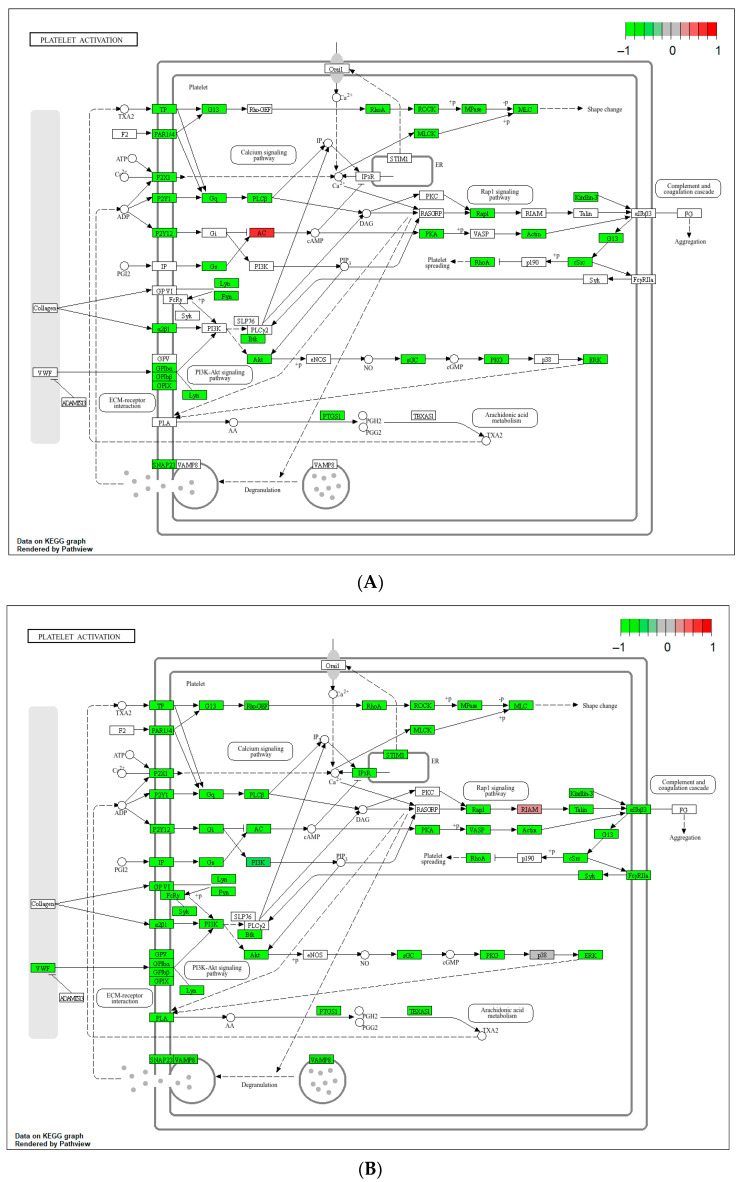
Platelet activation pathway (including calcium and Rap1 signaling pathways) with highlighted DEGs comparing (**A**) IRBD vs. CTRLs where an elevated number of DEGs was downregulated, and (**B**) DLB vs. CTRLs, where most of DEGs involved in this pathway were decreased in DLB. This massive downregulation of functional genes strongly suggests a major impairment of platelet activation, especially in DLB. Decreased genes are marked in green and increased genes in red. Solid arrows represent direct interactions between proteins; dashed arrows represent indirect or non-specified interactions between proteins.

**Figure A3:**
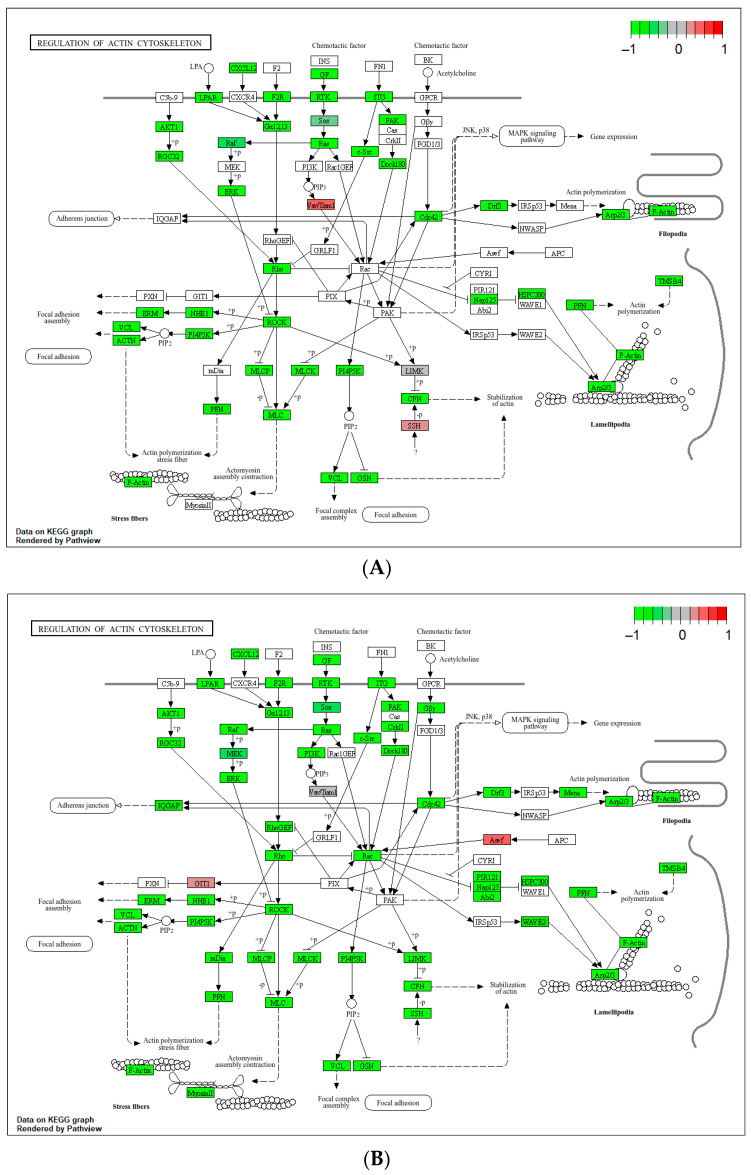
Regulation of actin cytoskeleton pathway with highlighted DEG comparing (**A**) IRBD vs. CTRLs with an elevated number of downregulated DEGs, and (**B**) DLB vs. CTRLs, where most of the genes involved in this pathway showed a decreased expression, suggesting a major impairment of actin cytoskeleton regulation starting in IRBD and being even more pronounced in DLB. Decreased genes are marked in green and increased genes in red. Solid arrows represent direct interactions between proteins; dashed arrows represent indirect or non-specified interactions between proteins.

**Figure A4:**
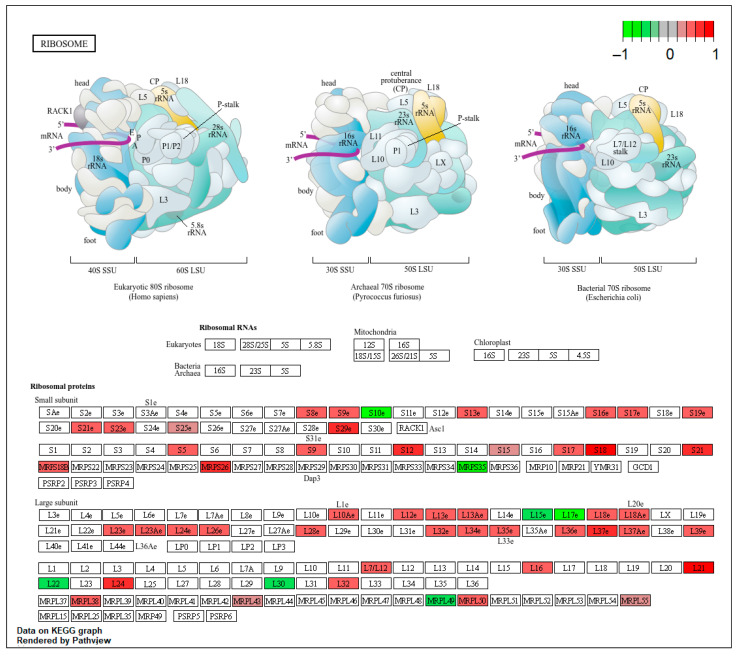
Ribosome components with highlighted DEGs comparing DLB vs. CTRLs, where most of the genes involved in the formation of ribosome showed an increased expression in the pathology group and where an enhancement of the pathway is expected. Decreased genes are marked in green and increased genes in red. In the ribosome representations, 5s rRNA is marked in yellow, 18s rRNA in blue, 28s rRNA in green and ribosomal proteins in grey.

## Data Availability

The data supporting the findings of this study will be deposited in a public repository upon completion of the full analysis of differences between disease groups. The datasets analyzed during the current study are available from the corresponding author on reasonable request.

## Abbreviations

The following abbreviations are used in this manuscript:

AD	Alzheimer’s disease
APP	amyloid precursor protein
Aβ	β-amyloid
CSF	cerebrospinal fluid
CTRLs	cognitively unaffected controls
DAT	dopamine transporter
DEA	differential expression analysis
DEGs	differentially expressed genes
DLB	Dementia with Lewy bodies
GDS	Global Deterioration Scale
GSEA	Gene Set Enrichment Analysis
GEO	Gene Expression Omnibus
GO	Gene Ontology
IRBD	idiopathic REM sleep behavior disorder
KEGG	Kyoto Encyclopedia of Genes and Genomes
LBD	Lewy body disorders
lncRNAs	long non-coding RNAs
MMSE	Mini-Mental State Examination
mtRNAs	mitochondrial RNAs
NGS	Next-Generation Sequencing
PD	Parkinson’s disease
PLT	Platelets
scaRNAs	small Cajal body-specific RNAs
snoRNAs	small nucleolar RNAs
snRNAS	small nuclear RNAs
SRA	Sequence Read Archive
UPDRS-III	Unified Parkinson’s Disease Rating Scale Part III

## Appendix A

### Appendix A.1

**Table A1:** LncRNAs specifically identified in DLB, AD, IRBD, PD and CTRLs.

	n	Ensembl Gene ID	External Gene Name	Chromosome	Start Position	End Position	Strand
DLB	1	ENSG00000237188		chr1	147172701	147295734	1
	2	ENSG00000289019		chr4	70853613	70903213	–1
	3	ENSG00000226281		chr6	6692737	6801186	–1
	4	ENSG00000287584		chr7	39621253	39623201	–1
	5	ENSG00000272293		chr8	450714	451343	–1
	6	ENSG00000284116		chr9	39931614	40106680	–1
	7	ENSG00000290769		chr9	133079900	133087355	1
	8	ENSG00000228886		chr13	45350323	45351350	1
	9	ENSG00000258803		chr14	56514331	56551309	1
	10	ENSG00000231439	*WASIR2*	chr16	22845	25191	1
	11	ENSG00000272884		chr17	7439506	7445966	1
	12	ENSG00000280800		chr21	8210384	8211306	–1
	13	ENSG00000284391		chrX	70427346	70435378	–1
AD	1	ENSG00000289474		chr2	148881726	148881841	–1
	2	ENSG00000242516	*LINC00960*	chr3	75672232	75742089	1
	3	ENSG00000290602		chr7	143810373	143818699	1
	4	ENSG00000289031		chr9	93566714	93568075	1
	5	ENSG00000288542		chr13	40469955	40611127	–1
	6	ENSG00000288855		chr14	50736300	50737517	–1
	7	ENSG00000259692	*LINC01418*	chr15	81610828	82013579	–1
	8	ENSG00000290383		chr16	18394972	18401925	–1
	9	ENSG00000185168	*LINC00482*	chr17	81303771	81311237	–1
	10	ENSG00000288235	*FAM106C*	chr17	16788879	16790501	1
	11	ENSG00000289172		chr20	45179818	45191491	1
	12	ENSG00000281181		chr21	8437629	8438551	–1
CTRLs	1	ENSG00000254154	*CRYZL2P-SEC16B*	chr1	177928788	178038007	–1
	2	ENSG00000273382	*TMEM167B-DT*	chr1	109087971	109090858	–1
	3	ENSG00000274769		chr2	61115787	61164825	1
	4	ENSG00000290614	*PRSS40A*	chr2	130570829	130584161	1
	5	ENSG00000289929		chr3	195635062	195652295	1
	6	ENSG00000288473		chr6	30908242	30926459	1
	7	ENSG00000290972		chr9	64369394	64412691	1
	8	ENSG00000289381		chr13	31796368	31814730	–1
	9	ENSG00000289049		chr14	101760727	101761485	–1
	10	ENSG00000291023		chr15	32406178	32434992	–1
	11	ENSG00000248101		chr19	36008638	36014235	–1
	12	ENSG00000268744		chr19	12379189	12401274	–1
	13	ENSG00000289298		chr19	41530216	41531859	–1
	14	ENSG00000288861		chr22	22757217	22759496	–1
IRBD	1	ENSG00000289062		chr1	152897765	152913138	–1
	2	ENSG00000289367		chr1	247937142	247937864	1
	3	ENSG00000291157		chr1	41302911	41306148	–1
	4	ENSG00000228363	*CHMP3-AS1*	chr2	86562070	86618766	1
	5	ENSG00000235070		chr2	226086623	226185651	–1
	6	ENSG00000226519	*LINC00390*	chr13	44094822	44161490	–1
	7	ENSG00000258694	*LINC02319*	chr14	52101631	52129852	–1
	8	ENSG00000290387	*SORD2P*	chr15	44825744	44884694	–1
	9	ENSG00000290674		chr16	21901552	21953031	–1
	10	ENSG00000261033	*SPECC1-DT*	chr17	20008051	20009234	–1
	11	ENSG00000176728	*TTTY14*	chrY	18772706	19077555	–1
	12	ENSG00000212856	*TTTY2B*	chrY	6406059	6462091	–1
	13	ENSG00000229308		chrY	4036335	4100619	1
	14	ENSG00000231535	*LINC00278*	chrY	3002887	3200509	1
	15	ENSG00000260197		chrY	19691941	19694606	–1
	16	ENSG00000288049		chrY	19744756	19759978	1
	17	ENSG00000289707		chrY	21138633	21257832	1
	18	ENSG00000290853		chrY	13703902	13916244	1
	19	ENSG00000291031	*BCORP1*	chrY	19455431	19567280	–1
	20	ENSG00000291033	*TXLNGY*	chrY	19567313	19606274	1
PD	1	ENSG00000225964	*NRIR*	chr2	6819463	6840464	–1
	2	ENSG00000189229		chr3	6490460	6736750	1
	3	ENSG00000251230	*MIR3945HG*	chr4	184843296	184855751	–1
	4	ENSG00000286274		chr5	129150677	129394114	1
	5	ENSG00000285492		chr6	159051674	159121510	–1
	6	ENSG00000173862		chr7	33725820	33729217	1
	7	ENSG00000289725		chr9	64411638	64469260	1
	8	ENSG00000290717	*ZNF658B*	chr9	39443589	39552802	–1
	9	ENSG00000286715		chr10	75592644	75628120	1
	10	ENSG00000290690		chr15	84398316	84422500	–1
	11	ENSG00000260280	*SLX1B-SULT1A4*	chr16	29455105	29464963	1
	12	ENSG00000290692		chr16	30204316	30209071	–1
	13	ENSG00000286288		chr20	1694082	1896406	–1
	14	ENSG00000291052	*ABCC13*	chr21	14236206	14338017	1

### Appendix A.2

**Table A2:** Distribution of minor RNAs in the five groups included in the study.

	n	scaRNA	snoRNA	snRNA	rRNA	mtRNA
DLB	55	10 (18%)	25 (45%)	11 (20%)	2 (4%)	7 (13%)
IRBD	36	8 (22%)	13 (36%)	7 (19%)	2 (6%)	6 (17%)
PD	58	9 (16%)	27 (46%)	18 (31%)	2 (4%)	2 (3%)
AD	75	12 (16%)	37 (49%)	20 (27%)	2 (3%)	4 (5%)
CTRLs	65	11 (17%)	30 (46%)	20 (31%)	2 (3%)	2 (3%)

### Appendix A.3

**Table A3:** List of minor RNAs found in PLTs in DLB, IRBD, PD, AD and CTRLs.

		DLB	IRBD	PD	AD	CTRLs
mtRNA	n	7	6	2	4	2
	1	MT-TL1	MT-TL1			
	2	MT-TV	MT-TV		MT-TV	
	3	MT-RNR2	MT-RNR2	MT-RNR2	MT-RNR2	MT-RNR2
	4	MT-TM	MT-TM			
	5	MT-TH			MT-TH	
	6	MT-TE	MT-TE			
	7	MT-RNR1	MT-RNR1	MT-RNR1	MT-RNR1	MT-RNR1
Ribozyme	n	2	2	2	2	2
	1	RMRP	RMRP	RMRP	RMRP	RMRP
	2	RPPH1	RPPH1	RPPH1	RPPH1	RPPH1
scaRNA	n	10	8	9	12	11
	1	SCARNA7	SCARNA7	SCARNA7	SCARNA7	SCARNA7
	2				SCARNA8	
	3	SCARNA6	SCARNA6	SCARNA6	SCARNA6	SCARNA6
	4	SCARNA5	SCARNA5	SCARNA5	SCARNA5	SCARNA5
	5	SCARNA10	SCARNA10	SCARNA10	SCARNA10	SCARNA10
	6	SCARNA12	SCARNA12	SCARNA12	SCARNA12	SCARNA12
	7	SCARNA13	SCARNA13	SCARNA13	SCARNA13	SCARNA13
	8			SCARNA21	SCARNA21	SCARNA21
	9					SCARNA3
	10	SCARNA1			SCARNA1	SCARNA1
	11	SCARNA16	SCARNA16	SCARNA16	SCARNA16	SCARNA16
	12	SCARNA2	SCARNA2	SCARNA2	SCARNA2	SCARNA2
	13	SCARNA4			SCARNA4	
snoRNA	n	25	13	27	37	30
	1				SNORA10	
	2				SNORA11	SNORA11
	3	SNORA12		SNORA12	SNORA12	SNORA12
	4	SNORA20			SNORA20	
	5			SNORA23	SNORA23	
	6	SNORA2C		SNORA2C	SNORA2C	SNORA2C
	7				SNORA33	
	8			SNORA37	SNORA37	SNORA37
	9				SNORA38B	
	10			SNORA48	SNORA48	
	11	SNORA53	SNORA53	SNORA53	SNORA53	SNORA53
	12	SNORA54		SNORA54	SNORA54	SNORA54
	13					SNORA57
	14		SNORA59B	SNORA59B	SNORA59B	SNORA59B
	15	SNORA5C			SNORA5C	
	16				SNORA62	
	17	SNORA63	SNORA63	SNORA63	SNORA63	SNORA63
	18				SNORA66	
	19	SNORA73A	SNORA73A	SNORA73A	SNORA73A	SNORA73A
	20	SNORA73B	SNORA73B	SNORA73B	SNORA73B	SNORA73B
	21	SNORA74A		SNORA74A	SNORA74A	SNORA74A
	22	SNORA74B		SNORA74B	SNORA74B	SNORA74B
	23	SNORA79B				
	24				SNORA7A	SNORA7A
	25	SNORA7B		SNORA7B	SNORA7B	SNORA7B
	26				SNORA8	SNORA8
	27	SNORA81	SNORA81	SNORA81	SNORA81	SNORA81
	28	SNORD10		SNORD10	SNORD10	SNORD10
	29	SNORD13		SNORD13	SNORD13	SNORD13
	30	SNORD15B	SNORD15B	SNORD15B	SNORD15B	SNORD15B
	31	SNORD17	SNORD17	SNORD17	SNORD17	SNORD17
	32	SNORD22			SNORD22	SNORD22
	33			SNORD33		
	34	SNORD3A	SNORD3A	SNORD3A	SNORD3A	SNORD3A
	35	SNORD3B-1	SNORD3B-1	SNORD3B-1	SNORD3B-1	SNORD3B-1
	36		SNORD3B-2	SNORD3B-2	SNORD3B-2	SNORD3B-2
	37	SNORD3C		SNORD3C	SNORD3C	SNORD3C
	38	SNORD89	SNORD89	SNORD89	SNORD89	SNORD89
	39	SNORD94		SNORD94		SNORD94
	40	SNORD97		SNORD97	SNORD97	SNORD97
	41		U3		U3	U3
snRNA	n	11	7	18	20	20
	1	RNU5A-1	RNU5A-1	RNU5A-1	RNU5A-1	RNU5A-1
	2	RNU5B-1		RNU5B-1	RNU5B-1	
	3	RNU4-1	RNU4-1	RNU4-1	RNU4-1	RNU4-1
	4	RNU4-2	RNU4-2	RNU4-2	RNU4-2	RNU4-2
	5				RNVU1-7	
	6			RNU1-28P	RNU1-28P	RNU1-28P
	7			RNU1-27P	RNU1-27P	RNU1-27P
	8		RNU1-1	RNU1-1	RNU1-1	RNU1-1
	9			RNVU1-18	RNVU1-18	RNVU1-18
	10			RNU1-2	RNU1-2	RNU1-2
	11			RNU1-4	RNU1-4	RNU1-4
	12					RNVU1-14
	13			RNU1-3	RNU1-3	RNU1-3
	14					RNU6ATAC
	15		RNU2-2P	RNU2-2P	RNU2-2P	RNU2-2P
	16	RNU6ATAC				
	17				RNVU1-2	RNVU1-2
	18	RNU2-2P				
	19	RNVU1-31		RNVU1-31	RNVU1-31	RNVU1-31
	20			RNVU1-29	RNVU1-29	RNVU1-29
	21	RNVU1-27	RNVU1-27	RNVU1-27	RNVU1-27	RNVU1-27
	22	RNU12		RNU12	RNU12	RNU12
	23	RNVU1-28		RNVU1-28	RNVU1-28	RNVU1-28
	24	RN7SK	RN7SK	RN7SK	RN7SK	RN7SK
